# Environmental toxins in neurodegeneration - a narrative review

**DOI:** 10.1186/s42466-025-00452-6

**Published:** 2025-11-17

**Authors:** Kristína Kulcsárová, Johannes Heinrich Alexander Piel, Eva Schaeffer

**Affiliations:** 1 Department of Neurology, P. J. Safarik University, Kosice, Slovak Republic; 2 Department of Neurology, L. Pasteur University Hospital, Kosice, Slovak Republic; 3 Department of Clinical Neurosciences, P. J. Safarik University, Kosice, Slovak Republic; 4 Department of Neurology, University Hospital Schleswig-Holstein, Campus Kiel and Kiel University, 24105 Kiel, Germany

**Keywords:** Neurodegeneration, Environmental toxins, Parkinson’s disease, Alzheimer’s disease, Amyotrophic lateral sclerosis, Pesticides, Heavy metals, Air pollution, Solvents

## Abstract

As the global incidence of neurodegenerative disorders rises at a rate beyond what can be attributed solely to population aging, the role of modifiable risk factors has come into research spotlight to inform preventive strategies. While many lifestyle interventions can be implemented at an individual level, addressing environmental pollutants that drive neurodegeneration requires a collective effort involving both public and political engagement. This narrative review summarizes current evidence on the role of selected environmental toxins—pesticides, solvents, air pollution, and heavy metals—in the development of Parkinson’s Disease, Alzheimer’s Disease, and Amyotrophic Lateral Sclerosis. Drawing from epidemiological and experimental studies, we highlight associations between these exposures and neurodegeneration, as well as potential converging pathophysiological mechanisms such as neuroinflammation and proteinopathy. Understanding these links may help inform public health measures aimed at reducing the burden of these diseases.

## Background

In recent decades, major financial investments have been made to advance disease-modifying pharmacological therapies for neurodegenerative diseases. Despite all the progress made, current developments are not yet sufficient to counter the rising prevalence of the most common neurodegenerative diseases worldwide. In view of these developments, the importance of preventive strategies, i.e. influencing modifiable risk factors in order to prevent the initial development and the acceleration of neurodegenerative diseases, has increasingly come to the fore. Several national and international organizations such as the World Health Organization (WHO) have emphasized the importance of prevention measures within the framework of specific programs, calls, and action plans. For Parkinson’s (PD) and Alzheimer’s disease (AD) in particular, the focus of preventive measures to date was primarily on measures that can be influenced individually such as lifestyle. However, it is becoming increasingly clear that also environmental toxins could play a decisive role in the development of neurodegenerative diseases. Although some environmental toxins have already been identified as relevant risk factors, such as pesticides for PD, the full spectrum of potentially modifiable environmental factors has not yet been exhausted. For example, the Lancet Commission has identified twelve modifiable risk factors that could reduce the prevalence of AD [1]. So far, these have mainly included personally influenceable risk factors such as low education, hearing loss, or smoking, while only one environmental toxin - air pollution - has been included in this risk constellation.

The clear identification of environmental toxins that are relevant for neurodegenerative diseases appears to be difficult due to the abundance of different toxins investigated and the resulting large number of studies. In order to draw the attention of both the public and political actors, it is therefore particularly important to identify specific environmental toxins whose evidence base is comprehensive enough to define them as clear targets for political action.

**Fig. 1:**
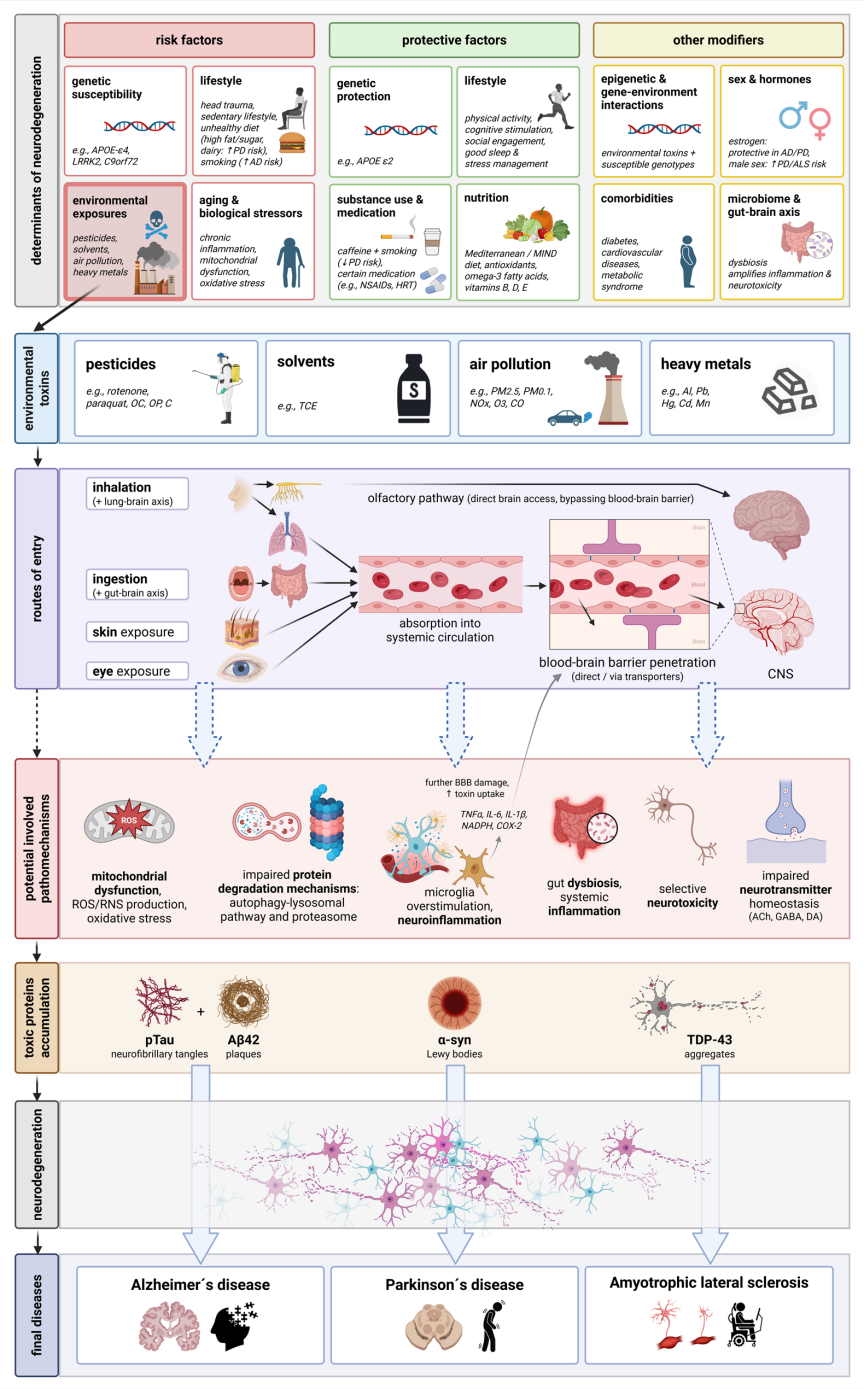
Pathological mechanisms of neurodegeneration following exposure to environmental toxins Dashed arrows show potential mechanisms by which environmental toxins could influence neurodegeneration. α-syn: α-synuclein; Aβ42: Amyloid β 42; ACh: acetylcholine; AD: Alzheimer’s Disease; Al: aluminium; ALS: amyotrophic lateral sclerosis; APOE: Apolipoprotein E; C: chlorines; C9orf72: chromosome 9 open reading frame 72; Cd: cadmium; CNS: central nervous system; CO: carbon monoxide; DA: dopamine; GABA: γ-Aminobutyric acid; Hg: mercury; HRT: hormon replacement therapy; LRRK2: Leucine-rich repeat kinase 2; MIND: Mediterranean-DASH Intervention for Neurodegenerative Delay diet; Mn: manganese; NSAID: Nonsteroidal anti-inflammatory drug; NOx: Nitrogen oxides; O3: ozone; OC: organochlorines; OP: organophosphates; Pb: lead; PM: particular matter; PD: Parkinson’s Disease; pTau: Hyperphosphorylated tau; RNS: reactive nitrogen species; ROS: reactive oxygen species; TCE: Trichlorethylene; TDP-43: TAR DNA-binding protein 43

The aim of this narrative review is to summarize the available studies on the most important potentially preventable environmental toxins and their contribution to the three most common neurodegenerative diseases (PD, AD, and amyotrophic lateral sclerosis, ALS), to present associations from epidemiological and clinical studies, and elucidate pathophysiological mechanisms (Fig. 1).

## Pesticides

### Clinical and epidemiological evidence

The term pesticides refers to substances or mixtures of substances intended to control insects, fungi, small animals, and plants, i.e. insecticides, fungicides, and herbicides. Pesticides are used in large quantities worldwide, especially in agriculture, and are absorbed by humans via inhalation, the skin, but also through food and water.

The association between pesticide exposure and PD is well established, supported by a large number of primary epidemiological case-control and cohort studies, as well as nearly 20 meta-analyses. These consistently report an increased risk of PD, with odds ratios (OR) ranging from 1.11 to 1.94 for pesticides in general, and from 1.24 to 2.19 for paraquat, one of the most strongly implicated compounds [2–17].

Despite this strong epidemiological evidence, several considerations remain before a causal relationship between specific environmental exposures and disease development—often decades later—can be firmly established. Pesticide exposure may be confounded by other environmental risk factors such as rural living, farming, well-water consumption [10], or increased incidence of head trauma in agricultural workers. These factors may act not only additively but also synergistically. For example, co-exposure to traumatic brain injury and paraquat has been shown to nearly triple PD risk compared to either exposure alone [18]. Genetic susceptibility may also modify the impact of pesticide exposure. Variants in genes such as the dopamine transporter [19], along with differences in pesticide metabolism, transport, mitochondrial vulnerability, oxidative stress response, and neuronal loss [20], may influence individual risk. Furthermore, although most meta-analyses treat pesticides as a homogenous group, important subclass differences exist—for instance, increased PD risk has been associated with insecticides and herbicides but not fungicides [21]. A recent study linked long-term exposure to 53 different pesticides with PD [22], though the strength of evidence for each agent varies and warrants further scrutiny. In addition, methodological issues such as inconsistent exposure assessments and recall bias may affect the observed associations in many epidemiological studies. While inferring causality from such studies is complex, Bradford Hill’s viewpoints (often referred to as BH criteria) offer a structured framework and a respected approach used in epidemiology. Although not all criteria—such as specificity or temporality—are conclusively met for every pesticide, the majority are - and as the BH framework is not intended as a rigid checklist, on the whole, the evidence supporting a causal relationship between pesticide exposure and PD is strong when viewed through this lens.

Importantly, beyond scientific consensus, policy changes reflect these findings: France recognized PD as an occupational disease among agricultural workers in 2012, and in March 2024, Germany formally adopted a scientific recommendation for a new occupational disease category: “Parkinson’s Disease caused by pesticides.” In parallel, the updated German guidelines for diagnosis and treatment of PD endorsed a major conceptual shift, abandoning the term “idiopathic” PD in recognition of the fact that many cases arise from genetic or environmental causes [23]. Moreover, pesticide exposure has been recognized as an independent prodromal PD risk factor in the Movement Disorder Society (MDS) research criteria for prodromal PD, both in its original and updated form [24].

Compared to the large number of cohort and case-control studies on PD, the epidemiological evidence for **AD** is considerably more limited. While several studies have found an association between pesticide exposure with (mild) cognitive impairment [25, 26] as well as AD specifically [9, 27–32], others have not [33, 34]. Moreover, the effect sizes are smaller than those reported for PD (OR 1.34, RR 1.50) [9]. A 2016 meta-analysis that specifically examined the link between cumulative pesticide exposure and the development of AD attributed these inconsistent findings to differences in study design, with a positive association observed only in cohort studies, but not in case-control studies [28]. Additionally, various methodological limitations have been discussed as contributing factors to the inconsistencies in epidemiologic research on pesticide exposure. These include reliance on self-reported or proxy-reported questionnaires, small sample sizes, imprecise exposure estimates, and short follow-up periods [35].

A distinctive feature of epidemiologic studies on the association between environmental toxins and **ALS** is the observation of an increased risk of ALS in veterans, which led to a detailed investigation of possible causative factors, including pesticides such as *Agent Orange* [36–38]. However, even independent of military service employment, pesticides show the most convincing evidence to date for environmental toxins regarding their association with ALS. Several systematic reviews and meta-analyses confirmed a significantly increased risk of ALS after exposure to pesticides in general, when analyzing specific compounds such as *organochlorines* in individuals with agricultural occupation [39–41]. However, contradictory results and potential influences of sex have also been reported here, with one meta-analysis only finding a positive association in men, but not in women [42].

### Pathomechanisms

Due to their small size and lipophilic nature, many pesticides can easily cross and disrupt the blood-brain barrier (BBB), leading to direct neurotoxic effects on the central nervous system (CNS) and shared molecular pathways of neurodegeneration such as neuroinflammation, induction of apoptosis and autophagy, oxidative stress, and mitochondrial dysfunction [43]. In addition, disease-specific pathophysiological pathways of individual pesticides were also investigated in various cell and animal models.

In this respect, the most impressive evidence of a direct pathophysiological association between pesticides and neurodegeneration has been found in recent years for **PD**. In particular *rotenone* and *paraquat* have been used in animal (mostly rodent) models to induce PD, thus serving as a proof-of-principle that environmental toxicants can have a direct role in selective degeneration of dopaminergic neurons in the Substantia nigra (SN) [44]. *Rotenone* acts mainly by inhibiting the function of mitochondrial complex I of the electron transport chain, resulting in excessive reactive oxygen species (ROS) production and mitochondrial dysfunction [45], to which dopaminergic neurons are particularly sensitive. Additional deleterious effects observed in PD animal models involve, beyond others, α-synuclein (α-syn) aggregation, changes in synaptic plasticity, impaired iron homeostasis, microtubule destabilization or proteasome inhibition [46–50]. Similarly, *paraquat* exerts its effects primarily through excessive ROS production [51], α-syn accumulation, but also proteasome inhibition and impaired dopamine catabolism [52]. In addition, synergistic effects of pesticides have also been investigated in these rodent models. Beyond others it has been observed that *maneb* can exacerbate the toxicity of *paraquat* by additionally inhibiting the enzymatic activity of mitochondrial complex III, and the systemic administration of both *paraquat* and *maneb* can induce a synergistic decrease of dopamine in the striatum and degeneration of SN [53]. In addition, *organochlorines* (e.g. *dieldrin*), *organophosphates* (e.g. *chlorpyrifos*), pyrethroids, and *glyphosate* were the most frequently studied toxins in terms of oxidative stress, proteasome dysfunction, neuroinflammation, α-synuclein aggregation and dopaminergic degeneration [54–57]. Beyond central neurotoxicity, peripheral effects of the pesticides on the microbiome and enteric nervous system (ENS) were described in recent years following the hypothesis of the microbiome-gut-brain axis as an important contributor to PD pathogenesis [58]. Rotenone exposure in cell and animal models impaired mitochondrial bioenergetics and activated inflammatory pathways in enteric glial cells, thereby exacerbating mitochondrial dysfunction and promoting pro-inflammatory processes that contribute to gut dysfunction [59], while paraquat triggered earlier expression of phosphorylated α-syn in the ENS of A53T mutant human α-syn transgenic mice [60]. Several rodent studies have shown that exposure to certain pesticides can trigger proinflammatory dysbiosis leading to intestinal inflammation, impaired intestinal barrier, and gastrointestinal dysfunction, followed by the formation of α-syn aggregates in the ENS and their transport through the vagus nerve to the brainstem [61].

In contrast, a variety of other heterogenous mechanisms have been proposed to explain cognitive decline and the development of **AD** following pesticide exposure [62]. In particular, exposure to *organophosphates*, *organochlorines*, and *carbamates* has been linked with cognitive decline and AD [28, 63–68]. These substances inhibit acetylcholinesterase (AChE) [69] and may promote tau protein hyperphosphorylation by overstimulating microglia, thereby triggering inflammatory and cytokine responses (including TNFα, IL6, IL-β, NADPH, COX-2), disrupting the BBB, and impairing amyloid elimination [63, 70–73]. Rodent studies have demonstrated increased phosphorylation of the microtubule-associated protein tau and microtubule-associated protein 2 following *organophosphate* exposure [74]. Furthermore, epidemiological studies have reported elevated serum levels of *dichlorodiphenyldichloroethylene* and *dichlorodiphenyltrichloroethane* in patients with AD [66–68], along with increased oxidative stress markers and reduced total antioxidant capacity [68]. In a rat model exposed to pyrethroids, oxidative stress was seen to promote glycogen synthase kinase 3 beta, which further accelerated the formation of neurofibrillary tangles [75]. Reduced activity of detoxifying enzymes such as paraoxonase 1 has been associated with AD and may contribute to the increased risk linked to *organophosphate* exposure [63]. Additionally, *organochlorines* disrupt GABA inhibition by blocking calcium-dependent sodium-potassium pumps and chloride channels, leading to hyperexcitation, mitochondrial dysfunction, and oxidative stress [43, 71]. However, despite the multitude of proposed pathways, findings from model organisms have limited translational relevance to humans, and current evidence is insufficient to establish a causal relationship between pesticides and the development of AD.

Possible interactions between genes and the environment were discussed for **ALS** and pesticides. In particular, it was investigated whether polymorphisms of the paraoxonase pathways (PON 1–3) increase susceptibility to ALS with pesticide exposure by impairing detoxification pathways. PON-1, for example, plays an important role in *organophosphate* metabolism [76–80]. However, studies to date brought conflicting results regarding the influence of PON-polymorphisms on ALS pathogenesis [81, 82]. In the course of increasing interest in pesticide-induced changes in the microbiome, it is of note that changes in the intestinal microbiome have been observed in ALS mice models and human studies [83]. For example, a reduction in *Akkermansia muciniphila* was observed in the SOD1 mouse model, and supplementation in turn improved the clinical symptoms of ALS in mice [84, 85]. A computational modelling study using human metagenomic and metatranscriptomic data indicated that *Akkermansia muciniphila* actively transcribes all genes of the shikimate pathway, suggesting that this bacterial species may be susceptible to the widely used pesticide glyphosate [86]. However, direct correlations have yet to be established, and the influence of pesticides on the microbiome and the associated disease mechanisms of neurodegenerative diseases, although desirable, has not yet been sufficiently researched.

### Summary

Although direct proof that pesticide exposure causes degeneration of the SN in humans is still lacking, mostly due to methodological and ethical limitations, epidemiological and experimental animal studies provide substantial indirect evidence that pesticides represent a significant modifiable risk factor for PD. The above-mentioned recognition of PD after pesticide exposure as an occupational disease, as well as the statement made in the WHO technical brief of 2023 to “ban pesticides […] which have been linked to PD” [38], are important first political steps that can not only help those already affected but also raise awareness among the general population. In contrast, while some epidemiologic studies show associations between pesticides and **AD** or **ALS**, pathophysiologic mechanisms are far less understood and there is a lack of comparable animal models that could provide a basis for understanding pesticide-related pathomechanisms.

## Solvents

### Clinical and epidemiological studies

Organic solvents such as alcohols or esters are widely used across various industries including paints, pharmaceuticals, cosmetics, and cleaning products, and have been implicated as potential risk factors for various neurodegenerative disorders.

Following several case, case-control and a twin study [11, 87, 88], solvents - in particular exposure to trichlorethylene (TCE) - have also been included in the MDS criteria for prodromal **PD** as relevant risk factors [24]. After many of these studies were conducted years to decades ago, a cohort study of over 150,000 veterans from the USA published in 2023 recently attracted attention. This study showed a significantly increased risk of developing PD or even prodromal PD symptoms in individuals exposed to TCE (stationed at Camp Lejeune) [89].

In contrast, there is insufficient epidemiological evidence to support link between solvents and **AD**, and recent data on this topic are limited. Chronic exposure to solvents, often occupational, can induce a condition known as *the painters’ syndrome* or *chronic solvent encephalopathy*, which is characterized by memory impairment, psychomotor slowing, depression, fatigue, sleep disturbances, and in severe cases, dementia [90]. While organic solvents have been linked to the development of other forms of dementia [27], particularly frontotemporal dementia, evidence linking solvent exposure to AD remains sparse [91, 92]. In a meta-analysis of occupational exposure and the development of AD, three studies conducted in the United States during the 1980s found no significant association of solvent exposure and AD risk [93]. A 1995 case-control study, however, suggested an increased risk of AD following solvent exposure [94]. More recently, a study reported global cognitive impairment in French men chronically exposed to gasoline, white spirit or cellulosic thinner [95]. Overall, clinical data remains insufficient to conclusively establish a causal relationship between solvents exposure and the development of AD.

The epidemiologic evidence for **ALS** is more robust. While it should be noted that several case-control and cohort studies did not investigate specific agents but solvents in general and therefore did not always find clear associations [96, 97], two meta-analyses, one from 2023, confirmed a significant association of solvent exposure to ALS development [91, 98].

### Pathomechanisms

The pathophysiological mechanisms of solvents have so far mainly been investigated in PD animal models exposed to *TCE*. As potentially disease-spanning pathomechanisms for neurodegeneration, pro-inflammatory effects, particularly microglial activation, could be observed [99, 100]. Moreover, other markers for oxidative stress such as 3-nitrotyrosine could be measured [101].

Similar to the neurotoxic properties of *rotenone*, the main effects of *TCE* are mediated by a reduction of mitochondrial complex I enzyme activity [87, 99, 100], which might again be specifically relevant for in this respect highly vulnerable SN neurons and, thus, the development of PD. The available rodent studies investigating neurotoxicity of *TCE* reported a selective and dose-dependent loss of dopaminergic neurons in the SN following *TCE* exposure [100–102]. In addition, α-syn inclusions were found in the SN and dorsal motor nucleus of the vagal nerve after oral administration of TCE in rats [87, 100–102].

However, compared to the extensive evidence from neurotoxic animal models of pesticides, PD animal studies on TCE are still limited to a small number, and animal models specifically investigating pathophysiological mechanisms of TCE and other solvents in **AD** and **ALS** are missing.

### Summary

Solvents, especially *TCE*, are still considered a relevant risk factor for PD, however, it must be noted that both the epidemiological and pathophysiological evidence is inferior to the clear results from pesticide research. While there are significant associations for ALS, at least from epidemiological studies, the evidence base for a link between solvents and AD is generally poor. However, considering the wider distribution of solvents and the multivariate possible exposure of humans via soil, water, but also as air pollution, it seems important to invest in further studies to show causal relationships with these neurodegenerative diseases.

## Air pollution

### Clinical and epidemiological studies

Air pollution comprises particulate matter (PM) of various sizes – coarse (*PM10*, < 10 μm), fine (*PM2.5*, < 2.5 μm) and ultra-fine (*PM0.1*, ≤ 0.1 μm) – as well as gaseous pollutants including ozone, nitrogen oxides (*NOx*), and carbon monoxide (*CO*), among others.

Air pollution is increasingly recognized as an emerging risk factor for PD, based on recent epidemiological studies. In the United States, maps depicting PD incidence show striking overlap with regions of elevated particulate air pollution [103], as well as with areas of high paraquat and *TCE* usage [104]. However, potential confounding factors—such as latency between exposure and disease onset, as well as migration patterns—must be considered. Traffic-related air pollution has been linked to increased PD risk in several observational studies across diverse regions, including Denmark, South Korea, and parts of the USA [105, 106]. Nonetheless, the evidence remains inconsistent for individual pollutants. For example, associations between *PM2.5* and PD risk were reported in several studies [103, 106–109], while other studies found no clear relationship [110, 111]. Similar variability has been seen with *PM10* [110, 112, 113] and *NO*_2_ [105, 110, 112, 114].

Palacios et al. [115] observed an almost equal number of studies reporting positive associations and null findings. Several recent meta-analyses have assessed various air pollutants—including *PM2.5*, *PM10*, *NO*_2_, *O*_3_, *CO*, coarse *PM*, *NO*_*x*_, and *SO*_2_—yet their conclusions remain contradictory. Reported associations with PD range from no or weak evidence for most pollutants to modest increases in risk for *PM2.5* (OR 1.01–1.34), *NO*_2_ (OR 1.01), and *ozone* (OR 1.01), and a more pronounced association for *CO* (RR 1.57) [116–123].

Air pollution is increasingly associated with impaired cognitive function and a potentially increased risk of **AD** and other forms of dementia [124–126]. In a range of case-control and cohort studies, summarized in in several systematic reviews and meta-analyses, particularly *PM2.5* has been linked to a higher risk of all-cause dementia, vascular dementia, and, to a lesser extent, to AD specifically [122, 127–130]. Longitudinal studies from Sweden reported an increased risk of AD (HR 1.38), vascular dementia (HR 1.47), and cognitive impairment following air pollution exposure [131–133]. Notably, the association between *PM2.5* exposure and AD risk appears to be independent of cerebrovascular damage [134]. Conversely, other longitudinal studies found an association of long-term air pollution exposure and vascular dementia or general cognitive decline, but not with AD specifically [135, 136]. A recent meta-analysis of 26 studies on all-cause dementia, including 12 focused on AD, proposed a RR of 1.37 for long term *PM2.5* exposure and dementias in general, with AD being the only dementia subtype significantly associated with *PM2.5* [137]. However, a more conservative estimate from another meta-analysis of 16 studies reported a HR of 1.04 for dementia risk after *PM2.5* exposure [138]. Potential biases, such as time trend and publication bias, must be considered [137, 138].

Several studies have also demonstrated significant associations with *NOx* [125, 129, 139]. In this respect it should be noted that *NOx* shows high spatial variability compared to *PM2.5*, contributing to higher variability in findings, which potentially explain the comparatively weaker evidence for *NOx* compared to *PM2.5* [138, 140]. Additionally, a possible gene-environment interaction has been described for *ozone* exposure, with carriers of the ApoE ε4 allele showing more rapid cognitive decline under high *ozone* levels [141]. The impacts of *PM10*, *CO*, and other components remain more controversial and less consistently reported [138, 140].

The link between **ALS** and air pollution was specifically discussed following the above-mentioned finding of an increased risk of ALS in veterans. Studies on the incidence of ALS among veterans have found a higher prevalence and cumulative incidence of ALS particularly after military service in the Air Force [142, 143]. Furthermore, some studies also found an increased risk among commercial pilots or flight attendants [144, 145]. A recent meta-analysis confirmed an increased risk for ALS risk in occupations with frequent contact with *diesel* and *jet exhaust* [146]. However, these correlations, derived purely from individual occupational sectors, remain a hypothesis and do not allow causality to be derived. For other components of air pollution, the evidence is still unclear. For example, a recent meta-analysis found no association with *PM2.5* or *PM10* [122].

### Pathomechanisms

The effects of *PM2.5*, which can enter the CNS via the BBB or directly via the olfactory neurons, have been particularly well studied, particularly in the context of the *lung-brain axis. PM2.5* and *PM0.1* can penetrate deep into the alveoli, initially causing pulmonary inflammation, whereupon the particles then enter the bloodstream to trigger systemic inflammation and - after overcoming the BBB – pathology in the CNS [147, 148]. Proposed pathogenetic mechanisms that may trigger converging pathways for neurodegenerative diseases, as investigated in animal studies, include oxidative stress and inflammatory pathways involving, among others, increased levels of IL-1α or TNF-α, as well as microglial activation [149–151]. In humans, both short- and long-term exposure to *PM2.5* have been related to an increase in inflammatory markers in the blood [152]. Although the *lung-brain axis* or direct uptake via the olfactory system appear to be the predominant pathways, influences on the *gut-brain axis* have also been described. In this respect changes in gut microbial diversity, as well as alterations of gut physiology, including gut inflammation and leakiness, have been observed [153, 154].

In addition, a compelling series of human autopsy studies by Calderón-Garcidueñas et al. investigated the effects of air pollution in Mexico City on the brains of young people who died suddenly. Chronic exposure to high levels of air pollution was associated with the presence of neuropathological markers linked to **AD** (hyperphosphorylated tau [pTau] and amyloid β 42 [Aβ42], ), **PD** (α-syn and Lewy neurites), and **ALS** (TAR DNA-binding protein 43 [TDP-43]) in the olfactory bulbs and other regions of the CNS - even in toddlers [155–157]. Furthermore, increased pathological protein accumulation in the CNS was accompanied by evidence of BBB disruption, oxidative stress, and inflammatory cell trafficking, all occurring as early as childhood [156].

Foreshadowing the subsequent discussion on heavy metals, particulate matter pollution may also act as a carrier for neurotoxic heavy metals originating from vehicles (e.g. *lead* from petrol) and industrial sources [158]. In one of the aforementioned studies from Mexico City, quadruple proteinopathy (the simultaneous presence of pTau, Aβ42, α-syn, and TDP-43) was observed alongside a high concentration of exogenous, heavy metal-rich nanoparticles, including those containing *iron* or *aluminium*. These findings suggest common pathogenic pathways underlying neurodegenerative diseases and point to a potential role of heavy metals in contribution to proteinopathy initiation from early childhood [155].

Moreover, disease-specific pathomechanisms have been explored in several cell and animal studies. In the context of **PD**, *diesel exhaust* particles have been shown to cause selective dopaminergic neurotoxicity in primary mesencephalic cultures and in exposed rats, primarily through mechanisms involving microglial activation and phagocytosis [159, 160]. *PM2.5* has been found to promote formation of α-syn fibrils with enhanced seeding activity, as well as induce mitochondrial dysfunction and oxidative stress in a transgenic α-syn A53T mouse model. Both intrastriatal injection and intranasal administration of *PM2.5* exacerbated α-syn pathology and dopaminergic neuronal degeneration [161]. *PM2.5* exposure has also been associated with motor impairments, disrupted white matter integrity, and dopaminergic neuronal loss in the olfactory and nigrostriatal circuits. It was shown to accelerate age-related demyelination and inflammation, and significantly increased TNF-α levels, leading to inflammatory changes in the fiber tracts of dopaminergic pathways in aged rats [162]. Similarly, long-term *PM10* exposure exacerbated motor impairments and dopaminergic neuronal loss in MPTP-induced PD mouse models, likely through increased pro-inflammatory mediators and microglial activation [163]. In contrast, exposure to ambient nano-particulate matter (*nPM*) had only a mild effect on α-syn propagation in the brain. However, combined exposure to *nPM* and α-syn synergistically downregulated Gria1 (glutamate receptor A1, involved in learning, memory, and olfactory processing) expression in both the olfactory bulb and cortex [164].

**AD**-specific pathology has been observed in two canine studies, which showed increased aggregation of β-amyloid plaques and tau-tangles in dogs exposed to urban air pollution [165]. In addition, the above-mentioned postmortem study of people from Mexico City found that APOE4 carriers were 6 to 13-times more likely to exhibit vascular amyloid in the olfactory bulb, neuronal amyloid accumulation, pTau neurofibrillary tangles, and neurites [157]. More recently, an impairment of the glymphatic system due to air pollution has been suggested as a potential mediator of neuroinflammation in AD development [166–168].

Apart from the cross-disease animal and post-mortem studies mentioned above, there are few studies specifically investigating the underlying pathomechanisms of **ALS**, although an increasing number of epidemiologic studies are linking ALS to air pollution.

### Summary

Overall, although evidence suggesting a link between air pollution and neurodegenerative diseases is growing, epidemiological studies still report inconsistent and sometimes contradictory results. This is mainly due to methodological issues, including the large number of different air pollution components measured, small sample sizes, timing, and methods for determining exposure and adjustment for other confounders and variables [125, 149]. The most convincing correlations for all three diseases so far have been for *PM2.5.* Moreover, the above-mentioned results from animal and post-mortem studies indicate that *PM2.5* in particular can trigger converging pathways of neurodegeneration, including systemic and consecutive neuro-inflammation, which, possibly in combination with individual predispositions, can then lead to pathological protein aggregation. Taken together, more specific studies with improved methods are urgently needed to target pathologically relevant aspects of air pollution.

## Heavy metals

### Clinical and epidemiological studies

Heavy metals are widely used in industry, agriculture, household products, and various technical applications, which can lead to localized or occupational accumulation in soil, air, and water. In certain regions or under specific conditions, this may result in concentrations that pose potential health and environmental risks. In addition to being classified as carcinogenic to humans, the possible neurotoxic effects of various heavy metals have also been intensively investigated in recent years.

Numerous epidemiological studies have explored the relationship between heavy metals and **PD**, with many reporting significant associations—particularly for *lead*, *mercury*, *copper*, *manganese*, *aluminium*, and *zinc* [169]. For example, individuals in industrial occupations exposed to *lead*, *copper*, and *manganese* for over 20 years had a 2–10-fold increased risk of PD [170]. However, overall evidence from recent meta-analyses remains inconclusive, as detailed in the following text - many report altered metal levels in PD patients, though findings vary and are often limited by methodological heterogeneity. *Iron* shows the strongest link to PD: postmortem studies consistently confirm elevated levels in the SN [171, 172]. In contrast, blood levels are inconsistent—some meta-analyses report decreases [173, 174], others increases [175] or no clear association [176]. Dietary *iron* intake may raise PD risk in Western populations [177]. *Copper* levels are generally lower in PD patients’ serum [174, 178], with similar findings in postmortem brain tissue [171], although one earlier meta-analysis found no difference [179]. Dietary *copper* intake was not associated with PD risk [177] (PMID: 26265293). *Zinc* levels are consistently reduced in serum and plasma [130, 174, 180], while hair levels may be elevated [174] and cerebrospinal fluid (CSF) findings are inconclusive due to small sample sizes, although a trend toward reduced levels was noted [181]. No significant association was observed with dietary *zinc* intake [177]. *Manganese* levels are elevated in serum but not in CSF [182]. Occupational *manganese* exposure, such as welding, has not been linked to PD, with confounding factors (e.g., smoking, healthy worker effect) suggested as explanations [183]. *Lead* demonstrates the most compelling evidence of an association with PD, particularly in the context of chronic or occupational exposure [9]. Studies assessing cumulative bone lead levels—a reliable indicator of long-term exposure due to lead’s decades-long half-life in bone—have consistently shown a strong association with increased PD risk [174, 184, 185]. Compared to studies evaluating only circulating metal concentrations, which may not accurately reflect long-term exposure, these findings position *lead* as the most robust environmental risk factor among the heavy metals studied.

In summary - regarding metals and PD, the most consistent findings are elevated brain *iron* and reduced serum *copper* and *zinc* levels. Among environmental risk factors, long-term *lead* exposure appears to have the strongest and most consistent association with PD. Nonetheless, further high-quality, prospective studies are essential to clarify causality and underlying mechanisms.

Neurotoxic and trace metals have been associated with the development of **AD.***Aluminium*, *mercury*, and *lead* were among the earliest suspected contributors. However, subsequent meta-analyses have yielded highly heterogenous results and pointed to potential publication and citation bias, underscoring the complexity and inconsistency of the finding in this area [186, 187]. More recently, research has increasingly focused on trace metals. Among these, the most consistent, yet weak, evidence exists for elevated *copper* levels in the blood of AD patients [178, 187–192]. However, the clinical significance of elevated *copper* in the development of AD remains more controversial. In contrast, findings for other trace elements are even more mixed. Some meta-analyses have reported decreased *zinc* levels in AD patients [191–193], while the evidence regarding *iron* remains inconclusive [187, 191]. The role of neurotoxic metals such as *aluminium*, *mercury* and *lead* also remains controversial. Epidemiologic studies and meta-analyses have suggested a possible association between chronic *aluminium* exposure and an increased risk of AD and reduced cognitive performance [194–197]. However, other meta-analyses have found no significant association [192, 198, 199]. Some meta-analyses have reported elevated *aluminium* levels in brain, serum, and CSF of patients with AD [200, 201]. One possible explanation for the heterogeneous results is individual variability in susceptibility to chronic *aluminium* exposure, potentially influenced by genetic factors [202, 203]. Similarly, studies investigating higher *mercury* levels have produced conflicting results. While higher *mercury* levels have been detected in blood samples in one meta-analysis [201], other meta-analyses have failed to confirm a significant association [186, 192]. Childhood *lead* exposure has been linked to increased risk of dementia and AD [204], but case-control studies have not provided clear in vivo evidence for *lead*’s role in AD development according to a systematic review [205] and four meta-analyses investigating heavy and trace metals have found no significant association of *lead* with AD risk [16, 186, 192, 201].

In **ALS**, some studies also focused on the measurement of heavy metals in biofluids (blood or CSF) and tissues. Most of these studies investigated measurable changes in *lead* concentrations, and several meta-analyses have found a link between elevated levels of *lead* in blood, CSF and, in some cases, bone tissue in ALS patients [9, 40, 206, 207]. Additionally, *mercury* was examined in some studies. Here too, increased concentrations were found in fingernails and hair [98, 208]. However, some inconsistencies were also found, e.g. reduced values in the blood of advanced-stage ALS patients [209]. Many other heavy metals have been investigated in epidemiological studies and clinical studies analyzing body fluids, however, sometimes found unclear or even inverse associations [206, 210, 211].

### Pathomechanisms

Multiple pathways mediate the accumulation of metals in the brain. For example *lead*, *cadmium*, and *manganese* can be transported via specific transporter proteins; *cadmium* and *manganese* may also enter the brain directly through the olfactory bulb [212]. The effects of chronic heavy metal exposure on neurodegenerative diseases are likely multifactorial, making it challenging to isolate individual mechanisms. In animal studies, *lead* exposure has been associated with inflammatory responses (e.g. increased IL-1 and TNF-α), accumulation of ROS, astrocyte damage, reduced metal clearance, oxidative stress, and excitotoxicity [158, 213, 214]. Similarly, microglial activation, elevated pro-inflammatory markers, mitochondrial dysfunction and oxidative stress-induced neuronal injury have been reported in relation to *aluminium* and *copper* exposure [215, 216].

While many neurotoxic effects of metals are non-specific, some evidence suggests disease-specific mechanisms. In the context of **PD**, excessive levels of some metals such as *manganese*, *iron*, *lead*, *mercury*, *aluminium*, and *cadmium* have been shown to induce injury in dopaminergic neurons [217–222]. The roles of other metals - such as *zinc*, *copper*, *selenium* – are more complex, with both beneficial and deleterious actions postulated in PD [223, 224]. The most widely implicated mechanisms of heavy metals in PD include mitochondrial dysfunction, oxidative stress, and neuroinflammation. In addition, metal-induced disruption of protein degradation pathways (e.g. proteasomal and autophagic systems) [221], as well as the promotion of α-syn aggregation and fibril formation has been demonstrated for several metal including *aluminium*, *iron*, *copper*, *lead*, *manganese*, and *mercury* [217, 225–229].

In **AD** pathophysiology, *aluminium* has been extensively investigated. Although animal and postmortem studies remain controversial, some evidence suggests that *aluminium* injections or dietary intake may induce β-amyloid aggregation and formation of neurofibrillary tangles [202]. *Mercury* has also been suspected of increasing the risk of AD, with in vitro and in vivo models showing similar changes [230, 231]. Experimental data on *lead* also suggest that chronic exposure may alter epigenetics, including DNA methylation, histone modification, and non-coding RNA activity [232, 233], as well as cause mitochondrial dysfunction, oxidative stress, and β-amyloid aggregation [234]. Finally, in vitro studies suggest that *copper* is an important trace metal in AD development as it has a high affinity for the copper binding site of the Aβ-peptide, which increases aggregation [235, 236].

For **ALS**, genetic susceptibilities and epigenetic mechanisms have been discussed as the basis for *lead*-induced neurotoxicity [232, 237–239]. In addition, it was observed in the mouse model that TDP-43 inclusions can occur [240]. Finally, accelerated onset of the ALS phenotype was found in a rodent model of ALS exposed to *mercury* [241].

### Summary

Despite the overwhelming number of studies, the current epidemiological evidence is still insufficient to establish a clear link between specific metal exposures and the risk of neurodegenerative diseases, as the results are very heterogeneous, and there are many methodological limitations, such as self-reporting [42, 242]. With regard to clinical studies, it should be noted that tissue and biofluid studies should be treated with caution, as they cannot determine causality, and many possible influencing factors, e.g. nutritional aspects, can also affect the direction of the correlation [209]. However, the constantly increasing number of in vivo studies could help to understand general, but also disease-specific pathomechanisms, which in the future could help to define the heavy metals with the greatest significance for neurodegenerative diseases.

## Conclusions

Many environmental toxins exhibit potential neurotoxic properties, and a number of epidemiological and pathophysiological studies indicate that some of these substances may contribute to the development of neurodegenerative diseases. However, assessing the impact of environmental toxins on neurodegenerative diseases remains challenging for several reasons. Firstly, measuring environmental exposure in humans is inherently complex, given the wide variations in duration, route, and intensity of exposure. Furthermore, the absence of standardized and validated methods to accurately quantify these exposures over extended periods adds to the difficulty. The evaluation is further complicated by the presumably long latency period—often spanning decades—between initial exposure and disease onset. Finally, a multitude of potential interaction factors must be taken into account, including gene-environment interactions as well as the interplay and interdependencies among various substances.

Following the unique pesticide animal models in PD, it becomes more and more evident that pesticides should be a target for policy interventions to counteract the development of neurodegenerative diseases. Although comparable animal models for AD and ALS are lacking, the overlapping pathophysiological pathways, which include the triggering of neuroinflammation and oxidative stress as well as specific, e.g. anticholinergic effects of various pesticides, make it likely that they are also relevant for these diseases.

Although measures have been taken in some countries to ban their use, many of them are still widely used as herbicides, fungicides or insecticides in agriculture and aquaculture due to their low cost and effectiveness against pests. However, several useful approaches have been identified. Firstly, the use of relevant pesticides should be reduced and replaced by safer alternatives such as biopesticides and genetically modified organisms. In addition, protective measures, such as the use of personal protective equipment or new approaches to treating poisoning, should be emphasized in cases where pesticide exposure remains unavoidable.

As the impaired gut–brain axis is recognized as an important contributor to PD pathogenesis [58] and several pesticides have been shown to induce changes in the ENS and promote dysbiosis—thereby leading to microbiome–gut–brain axis dysfunction [61], targeting this axis may offer novel preventive and therapeutic opportunities. Strengthening a healthy microbiome through general lifestyle interventions, particularly dietary modifications [243–245] and exercise [246], has shown promise both in the general population and in the context of PD. In addition, more targeted microbiota-modulating strategies—such as the use of probiotics, prebiotics, antibiotics, or microbial metabolite-based interventions—have demonstrated beneficial effects in animal models and some human studies. For example, randomized controlled trials (RCTs) in PD patients have shown that probiotic supplementation significantly improved the gene expression of inflammatory mediators, including IL-1 and TNF-α [247], and had favorable effects on MDS-UPDRS scores and certain metabolic profiles [248]. In the context of pesticide-related exposure, probiotics have shown beneficial effects on mitochondrial function and energy metabolism in toxin-induced animal models [249], and have been shown to protect nigral dopaminergic neurons from MPTP- and rotenone-induced neurotoxicity [250]. Therefore, in specific contexts—such as conditions potentially associated with pesticide exposure—more targeted interventions using prebiotics, probiotics, or postbiotics may hold therapeutic potential. However, further research is needed to confirm their efficacy and safety.

For the other most frequently discussed environmental toxins (solvents, air pollution, and metals), the assessment of the evidence is made considerably more difficult by the many methodological issues discussed above. Many further research steps are ahead of us to build any recommendations on a solid scientific basis - such as developing reliable markers of exposure. For all environmental toxins, epidemiological studies often do not differentiate between specific agents or consider synergistic toxicity, e.g., metals that increase the toxicity of other metals or pesticides. Finally, gene-environmental interactions need to be better understood in order to elucidate how individual susceptibilities to environmental toxins arise from common cross-disease mechanisms and how this can be used as a basis for the targeted protection of individuals at-risk. Finally, a great challenge for political action is the considerable time lag between early exposure to environmental toxins and the onset of manifest neurodegenerative diseases, which can take decades. Even if effective measures were taken immediately, it could take years before a gradual decline in neurodegenerative diseases is observed.

However, even if toxic effects are not disease-specific, a small increase in risk can have a large impact on a global scale if concentrated environmental toxins in densely populated urban areas affect hundreds of millions of people. As an example, the WHO recommends keeping annual *PM2.5* levels below 5 µg/m³. However, *PM2.5* levels have been reported to exceed this threshold by up to ten times, even in Western countries where they more than double the recommended limits. The detailed analysis of possible pathophysiological links to neurodegenerative effects of these widespread environmental toxins must therefore become a priority.

## Data Availability

Not applicable (review article).

## Abbreviations

α-syn: α-synuclein
Aβ-42: Amyloid β 42
AChE: Acetylcholinesterase
AD: Alzheimer’s disease
ALS: Amyotrophic lateral sclerosis
BBB: Blood brain barrier
CNS: Central nervous system
CO: Carbon monoxide
ENS: Enteric nervous system
MDS: Movement Disorder Society
NOx: Nitrogen oxides
PD: Parkinson’s disease
PM: Particular matter
PON: Paraoxonase
pTau: Hyperphosphorylated tau
ROS: Reactive oxygen species
SN: Substantia nigra
TCE: Trichlorethylene
TDP43: TAR DNA-binding protein 43
WHO: World Health Organization
